# Persistent vessel wall enhancement and progression of cerebral microbleeds in CADASIL: a case report

**DOI:** 10.3389/fradi.2026.1782068

**Published:** 2026-04-02

**Authors:** Wenjuan Xu, Chao Zhang, Xiaomin Liu, Xiaoyu Zhang

**Affiliations:** 1 Department of General Practice, The First Affiliated Hospital of Shandong First Medical University & Shandong Provincial Qianfoshan Hospital, Jinan, China; 2 Department of Radiology, The First Affiliated Hospital of Shandong First Medical University & Shandong Provincial Qianfoshan Hospital, Jinan, China; 3 Department of Neurology, The First Affiliated Hospital of Shandong First Medical University & Shandong Provincial Qianfoshan Hospital, Jinan City, China

**Keywords:** case report, cerebral autosomal dominant arteriopathy with subcortical infarcts andleukoencephalopathy, cerebral microbleeds, stroke, vessel wall enhancement

## Abstract

Cerebral autosomal dominant arteriopathy with subcortical infarcts and leukoencephalopathy (CADASIL) is the most prevalent hereditary cerebral small vessel disease (cSVD), primarily caused by pathogenic variants in the *NOTCH3* gene. Neuroimaging features, including white matter hyperintensities (WMH), lacunar infarcts, and cerebral microbleeds (CMBs), typically progress with disease advancement. However, the pathophysiological mechanisms underlying this neuroimaging progression remain poorly understood. Here we present a patient with a *NOTCH3* mutation who exhibited persistent vessel wall enhancement on serial high-resolution vessel wall MRI (VWMRI), alongside progression of CMBs. This finding supports a critical role of blood-brain barrier (BBB) disruption in CADASIL pathophysiology. In conclusion, persistent intracranial vessel wall enhancement may be observed in patients with CADASIL. Further studies are needed to investigate the relationship between this imaging biomarker and clinical as well as neuroimaging outcomes.

## Introduction

Cerebral small vessel disease (cSVD) is the widely accepted term for a heterogeneous group of diseases, which is a major cause of ischemic stroke and dementia (1). Genetic mutations play an important role in the pathogenesis of cSVD. In hereditary cSVD, cerebral autosomal dominant arteriopathy with subcortical infarcts and leukoencephalopathy (CADASIL) is the most prevalent, which is primarily associated with *NOTCH3* mutations (2). Typical clinical manifestations of CADASIL include migraine with aura, recurrent ischemic strokes, and progressive cognitive impairment.

On brain magnetic resonance imaging (MRI), patients with CADASIL commonly exhibit symmetrical white matter hyperintensities (WMH), lacunar infarcts, cerebral microbleeds (CMBs) and brain atrophy. Anterior temporopolar and external capsule WMH may serve as a valuable diagnostic marker for differentiating CADASIL from other cSVD subtypes. Disease progression is often accompanied by worsening WMH burden, increasing CMBs and lacunar infarcts, which appear to correlate with clinical severity. Nevertheless, the pathophysiological mechanisms underlying neuroimaging progression in CADASIL remain poorly elucidated. Proposed hypotheses include chronic cerebral hypoperfusion, blood-brain barrier (BBB) dysfunction, and impaired cerebrovascular reactivity (2). However, direct supporting evidence remains relatively limited. In support of the BBB disruption hypothesis, only a previous high-resolution vessel wall MRI (VWMRI) study demonstrated intramural patchy gadolinium enhancement within subcortical and leptomeningeal vessels (3). Here we present a patient with a *NOTCH3* mutation demonstrating persistent vessel wall enhancement on serial high-resolution vessel wall MRI VWMRI, concurrent with progression of CMBs.

## Case report

A 55-year-old woman, with a history of hypertension, ischemic stroke and intracranial hemorrhage, was admitted to hospital because of one month of numbness in her limbs and lips on May 15, 2018. She also complained of memory loss and blurred vision. On admission, neurologic examination was normal except for declined recent memory. Her Montreal Cognitive Assessment (MoCA) score was 25 and National Institutes of Health Stroke Scale (NIHSS) score was 0. Blood tests revealed elevated thyroglobulin antibodies and thyroid peroxidase antibody, indicating Hashimoto's thyroiditis. An autoimmune screen was positive for antinuclear antibody and anti-Jo-1 antibody. Blood routine examination, glucose, serum lipid, liver and renal function, coagulation, syphilis and HIV tests were normal. Cerebrospinal fluid (CSF) analysis was normal except for an elevated IgG level (IgG = 51.7 mg/l). Findings of clinical and laboratory work-ups were negative for central nervous system infection and neoplasm.

Electromyography was performed to investigate cause of numbness in her limbs and lips. Results showed bilateral median nerve injury. Decreased sensory nerve conduction velocity on the left median nerve was noted. Prolonged motor nerve latency along with decreased compound muscle action potential (CMAP) amplitude on the right median nerve were recorded. Sensory nerve action potentials (SNAP) were undetectable on the right median nerve. (Table 1) Needle electromyography results are normal.

**Table 1:** Electrophysiological data at admission.

Nerve conduction study parameters	Left median	Left ulnar	Right median	Right ulnar
DL (ms) dis./prox.	4.1/8.1	1.8/5.9	6.8/11.5	1.9/5.6
CMAP (mV) dis./prox.	10.6/10.1	11.5/10.3	3.0/2.8	14.4/14.0
MCV (m/s)	52.5	57.3	43.6	56.8
F-latency (ms)	26.2	26.1	30	25.1
F-frequency (%)	90	100	100	90
SNAP (*μ*V)	26.9	23.8	N.D.	13.9
SCV (m/s)	36.7	57.9	N.D.	51.7

DL, distal latency; CMAP, compound muscle action potential; MCV, motor conduction velocity; SNAP, sensory nerve action potential; SCV, sensory conduction velocity; N.D., not detected.

**Figure 1:**
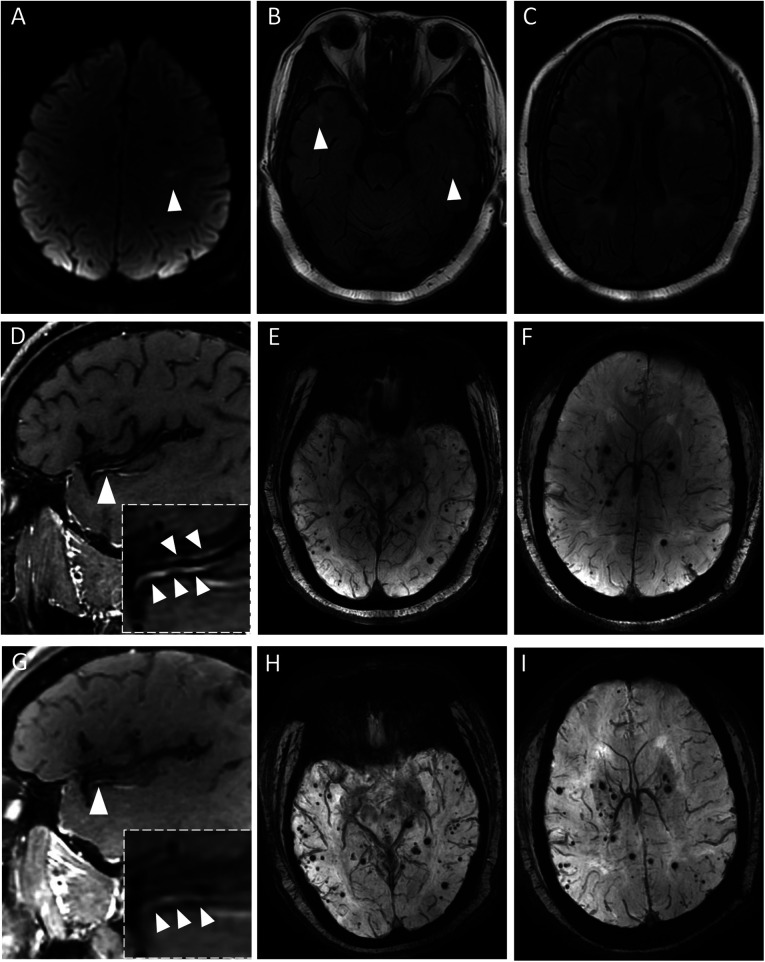
Neuroimaging features at admission and serial high-resolution vessel wall MRI (VWMRI) findings. **(A)** DWI hyperintensity in the left centrum semiovale showed acute ischemic stroke at admission. **(B,C)** white matter hyperintensities (WMH) were observed in bilateral temporal lobes, subcortical and periventricular regions on T2-FLAIR. **(D)** VWMRI revealed homogeneous, concentric vascular wall enhancement with thickening along a branch of the right MCA. **(E,F)** Cerebral microbleeds (CMBs) in the frontal lobe, parietal lobe, temporal lobe, occipital lobe at admission on susceptibility-weighted imaging (SWI). **(G)** Follow-up VWMRI at 14 months demonstrated persistent enhancement in the same branch of the right MCA. **(H,I)** Number of CMBs increased in the follow-up SWI.

Brain MRI revealed diffusion weighted imaging (DWI) hyperintensity in the left centrum semiovale, indicating acute ischemic stroke. (Figure 1) Multiple WMH were located in the temporal lobe, bilateral subcortical and periventricular regions (The total Fazekas score was 5, with a periventricular score of 3 and a subcortical score of 2). Susceptibility-weighted imaging (SWI) demonstrated 66 CMBs in the frontal lobe, parietal lobe, temporal lobe, occipital lobe and basal ganglia. Cerebellar hemosiderin deposition was associated with a history of intracranial hemorrhage. Several lacunes were observed within WMH-affected areas. Conventional MR angiography showed normal intracranial arteries. To evaluate vessel wall lesions and enhancement in the meninges and parenchyma, VWMRI was performed on the third day after admission. The scans demonstrated homogeneous and concentric vessel wall enhancement with thickening along the branches of the right middle cerebral artery and the left posterior cerebral artery. The enhancement was milder than the pituitary stalk, consistent with mild to moderate enhancement. No enhancement was detected in the leptomeninges or the brain parenchyma. The patient was treated with aspirin and statin according to the guideline for acute ischemic stroke. Methylprednisolone (40 mg/day for 2 weeks, 20 mg/day for ten days and 10 mg/day for ten days) was prescribed for initial suspicion for vasculitis and antihypertensive drugs was used to control her hypertension.

To evaluate the possibility of hereditary cSVD, genetic testing of a peripheral blood sample was performed. One month later, results identified a *NOTCH3* gene mutation in exon 28: c.5171_–_5172del, p. (Thr1724fs). Therefore, this finding confirmed the diagnosis of CADASIL. Aspirin, statin and antihypertensive drugs were prescribed. During one-year follow-up period, her MoCA score was 24, and no new cerebral events occurred. Follow-up VWMRI in the same machine at 14 months demonstrated persistent vascular wall enhancement (mild to moderate) and thickening in the middle and posterior cerebral artery branches. No significant progression of WMH was observed (Total Fazekas score = 5, with a periventricular score of 3 and a subcortical score of 2), while the number of CMBs increased from 66 to 81. (Figure 1)

## Discussion

As the most prevalent hereditary cSVD, CADASIL typically presents on MRI with symmetrical WMH, lacunar infarcts, CMBs, and brain atrophy. However, studies focusing on intracranial vessel wall changes in CADASIL patients remain limited. In the present case, longitudinal follow-up documented persistent vessel wall enhancement concurrent with the progression of CMBs.

Compared to conventional luminal imaging techniques for intracranial arteries, VWMRI has the potential to directly visualize vessel wall lesions. Vascular wall enhancement seen on VWMRI is considered to be associated with inflammatory processes, increased BBB permeability, or neovascularization in the vessel wall (4). CADASIL is a non-atherosclerotic and non-amyloid diffuse angiopathy primarily involving small to medium-sized arteries, capillaries, and small veins. The pathological characteristics of CADASIL include a vasculopathy manifested by the degeneration of vascular smooth muscle cells (VSMCs) and deposition of granular osmiophilic material (GOM) along the plasma membrane of VSMCs and pericytes, which may compromise BBB permeability (2). Assessed by diffusion-prepared pseudo-continuous arterial spin labeling (DP-pCASL), the BBB water exchange rate was decreased in patients with CADASIL, suggesting BBB dysfunction (5). A previous case report also described intramural patchy gadolinium enhancement in the subcortical and leptomeningeal arteries of a CADASIL patient on VWMRI (3) In our case, we observed similar vessel wall enhancement, further supporting the hypothesis that BBB disruption plays a critical role in the pathophysiology of CADASIL. The differential diagnosis includes cerebral amyloid angiopathy (CAA) and hypertensive arteriopathy, as both conditions may present with cerebral microbleeds and intracranial vessel wall enhancement on VWMRI. However, in hypertensive arteriopathy, microbleeds are predominantly located in the basal ganglia, a finding not consistent with our patient. Moreover, CAA is unlikely according to the Boston criteria version 2.0, which require the absence of any deep hemorrhagic lesions for diagnosis (6). In the absence of pathological evidence for our patient, we cannot completely rule out the possibility of comorbidities such as CADASIL, hypertensive arteriopathy, and CAA. Additionally, Hashimoto's encephalopathy was not considered due to the absence of behavioral abnormalities and hyperintensity on DWI (7).

More importantly, this study is the first to report persistent vessel wall enhancement at the one-year follow-up, which coincided with the progression of CMBs. Unfortunately, the underlying mechanisms of persistent vessel wall enhancement and the progression of CMBs remain largely unknown. CMBs are found in 31%–60% of the CADASIL patients, occurring more frequently in deep gray matter but also in lobar regions (8). In mouse model, inflammation-induced CMBs have been linked to BBB disruption (9). Furthermore, cerebral iron burden has been associated with regional BBB permeability in CADASIL patients, supporting a close relationship between BBB impairment and CMBs (10). Based on these findings, we hypothesize that persistent vessel wall enhancement reflects increased BBB permeability, which may be associated with a higher incidence of CMBs in CADASIL. This hypothesis is supported by observations in CAA, where patients exhibiting vessel wall enhancement presented with numerous CMBs, whereas those without enhancement had few or none (11). Other predictors of neuroimaging progression in CADASIL had been identified in previous studies. A longitudinal study demonstrated that lower baseline cerebrovascular reactivity (CVR) was associated with a greater increase in WMH volume (12). Unfortunately, WMH volume was not calculated in our study. Additionally, patients with high risk *NOTCH3* variants (EGFr domains 1–6, 8,11,26) have been shown to exhibit significantly faster progression in major clinical and neuroimaging outcomes compared to those with medium-risk *NOTCH3* variants (13). Therefore, cohort studies are required to investigate whether persistent vessel wall enhancement can serve as a predictor for the progression of neuroimaging abnormalities in CADASIL, which may offer novel insights into its pathogenesis.

Only a few studies have reported peripheral neuropathy in CADASIL patients (14, 15). The major electrophysiological manifestation in CADASIL patients is sensorimotor polyneuropathy (14, 16). In our case, patient exhibited bilateral median nerve injury, consistent with previous reports. Pathological studies revealed reduced nerve fiber densities in sural nerve biopsies and decreased intraepidermal nerve fiber density in skin biopsies (17). Electron microscopic examination revealed the deposition GOM in the wall of endoneurial small vessels in the peripheral nerve (15). These pathological changes likely contribute to vascular dysfunction and chronic low-grade chronic ischemia in peripheral nerves, ultimately resulting in clinical symptoms. It should be noted that the median nerve involvement in our patient appeared unrelated to the presence of antinuclear antibodies and anti-Jo-1 antibodies, given the absence of autoimmune disease manifestations**.**

## Conclusion

Persistent intracranial vessel wall enhancement may be observed in patients with CADASIL, suggesting a critical role of BBB disruption in CADASIL pathophysiology. Further studies are needed to investigate the relationship between such enhancement and clinical and neuroimaging outcomes.

## Data Availability

The original contributions presented in the study are included in the article/Supplementary Material, further inquiries can be directed to the corresponding author/s.
